# Supplementary material for: Nonideality‐Aware Training for Accurate and Robust Low‐Power Memristive Neural Networks
Source: Adv Sci (Weinh). 2022 May 4;9(17):2105784. doi: 10.1002/advs.202105784 (PMC9189678; doi:10.1002/advs.202105784)
Supplement: Supplementary file 1 — Supporting Information [file ADVS-9-2105784-s001.pdf]

ADVANCED SCIENCE
Open Access

Supporting Information

for *Adv. Sci.*, DOI 10.1002/advs.202105784

Nonideality-Aware Training for Accurate and Robust Low-Power Memristive Neural Networks

Dovydas Joksas, Erwei Wang, Nikolaos Barmpatsalos, Wing H. Ng, Anthony J. Kenyon, George A. Constantinides and Adnan Mehonic**

Tables

ID	Nonideality	G_{off} (S)	G_{on} (S)	Parameters
1	Low I-V nonlinearity in SiO_x	6.901×10^{-4}	3.451×10^{-3}	See Figure 2
2	High I-V nonlinearity in SiO_x	5.248×10^{-7}	2.624×10^{-6}	See Figure 2
3	Stuck at G_{off}	-	-	$P(\text{stuck}) = 0.05$
4	Stuck at G_{on}	-	-	$P(\text{stuck}) = 0.05$
5	Stuck Ta/HfO_2 devices	4.364×10^{-5}	9.782×10^{-4}	$P(\text{stuck}) = 0.101$
6	More uniform D2D variability	-	-	$\sigma\|_{R=R_{\text{off}}} = \sigma\|_{R=R_{\text{on}}} = 0.25$
7	Less uniform D2D variability	-	-	$\sigma\|_{R=R_{\text{off}}} = 0.5$, $\sigma\|_{R=R_{\text{on}}} = 0.05$
8	High-magnitude D2D variability	-	-	$\sigma\|_{R=R_{\text{off}}} = \sigma\|_{R=R_{\text{on}}} = 0.5$

Table S1: All nonidealities utilized in the simulations. For mappings onto crossbar arrays, G_{off} and G_{on} of the nonidealities were used. Where these values were not available (because the models did not use experimental data), they were borrowed from nonideality 2.

ID	Nonideality IDs	Notes	Figures
1	-	Mapping from Equation (5) and standard validation.	4a,d, 5, 6c, 8
2	1	-	4b, 5, 8
3	1	Regularized.	4c, 5, 8
4	2	-	4e, 5, 6, 8
5	2	Regularized.	4f, 5, 8
6	6	Mapping from Equation (4).	7a,i
7	6	Mapping from Equation (5).	7b,i
8	6	-	7c,i, 8
9	6	Regularized.	7d,i, 8
10	7	Mapping from Equation (4).	7e,j
11	7	Mapping from Equation (5).	7f,j
12	7	-	7g,j, 8
13	7	Regularized.	7h,j, 8
14	8	-	8
15	3	-	8
16	5	-	8
17	2, 4	-	8

Table S2: Training setups. Unless stated otherwise, the networks used double weights, regularization was not applied and memristive validation was used.

ID	Nonideality IDs	Notes	Figures
1	-	Mapping from Equation (5).	8
2	1	-	4a–c, 5, 8
3	2	-	4d–f, 5, 6, 8
4	6	Mapping from Equation (4).	7i
5	6	Mapping from Equation (5).	7i
6	6	-	7i, 8
7	7	Mapping from Equation (4).	7j
8	7	Mapping from Equation (5).	7j
9	7	-	7j, 8
10	8	-	8
11	3	-	8
12	5	-	8
13	2, 4	-	8

Table S3: Inference/test setups. This includes both the figures where the networks were evaluated on the test set after they had been fully trained and the figures in which test set performance was evaluated during training. Unless stated otherwise, the networks used double weights.

Name	Training setup ID	Inference setup ID
Ideal	1	1
Low I-V nonlin. [SiO_x]	2	2
Low I-V nonlin. [SiO_x] (reg.)	3	-
High I-V nonlin. [SiO_x]	4	3
High I-V nonlin. [SiO_x] (reg.)	5	-
Stuck at G_{off}	15	11
Stuck [Ta/HfO_2]	16	12
More uniform D2D var.	8	6
More uniform D2D var. (reg.)	9	-
Less uniform D2D var.	12	9
Less uniform D2D var. (reg.)	13	-
High I-V nonlin. [SiO_x] + stuck at G_{on}	17	13
High D2D var.	14	10

Table S4: Training and inference setups used in Figure 8.

Figures

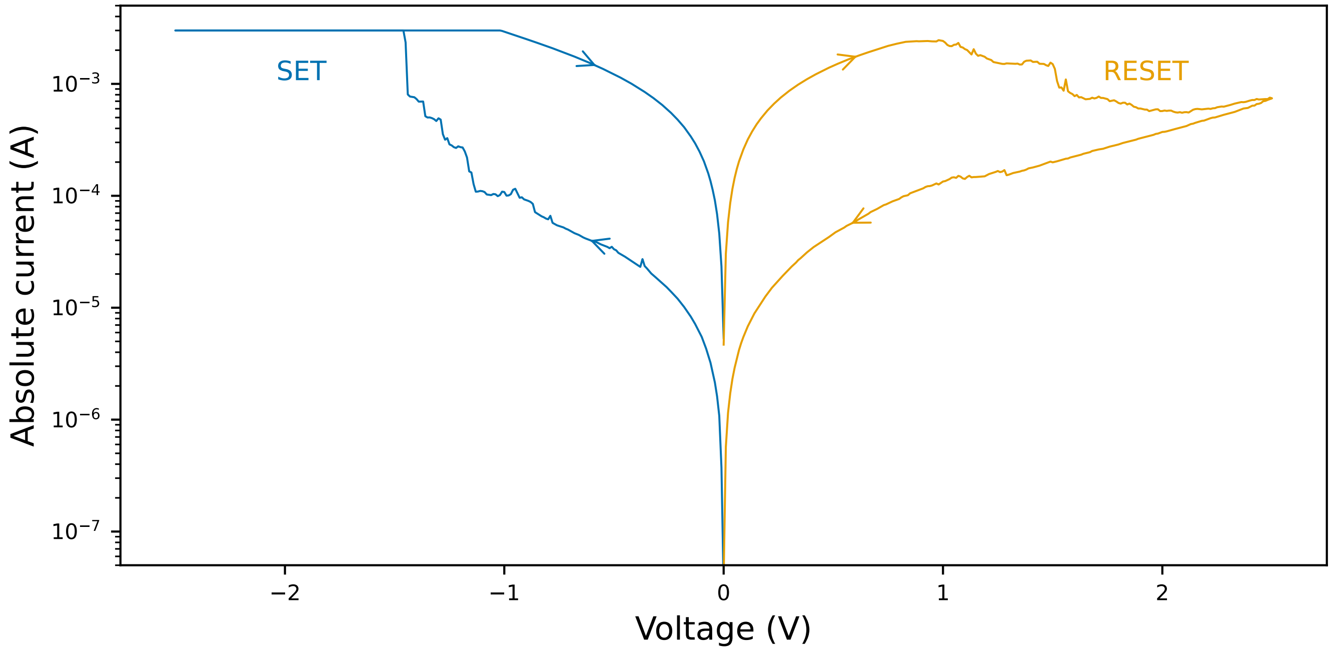

Figure S1: Typical resistance switching behavior of the SiO_x device. One SET and one RESET sweep are shown.

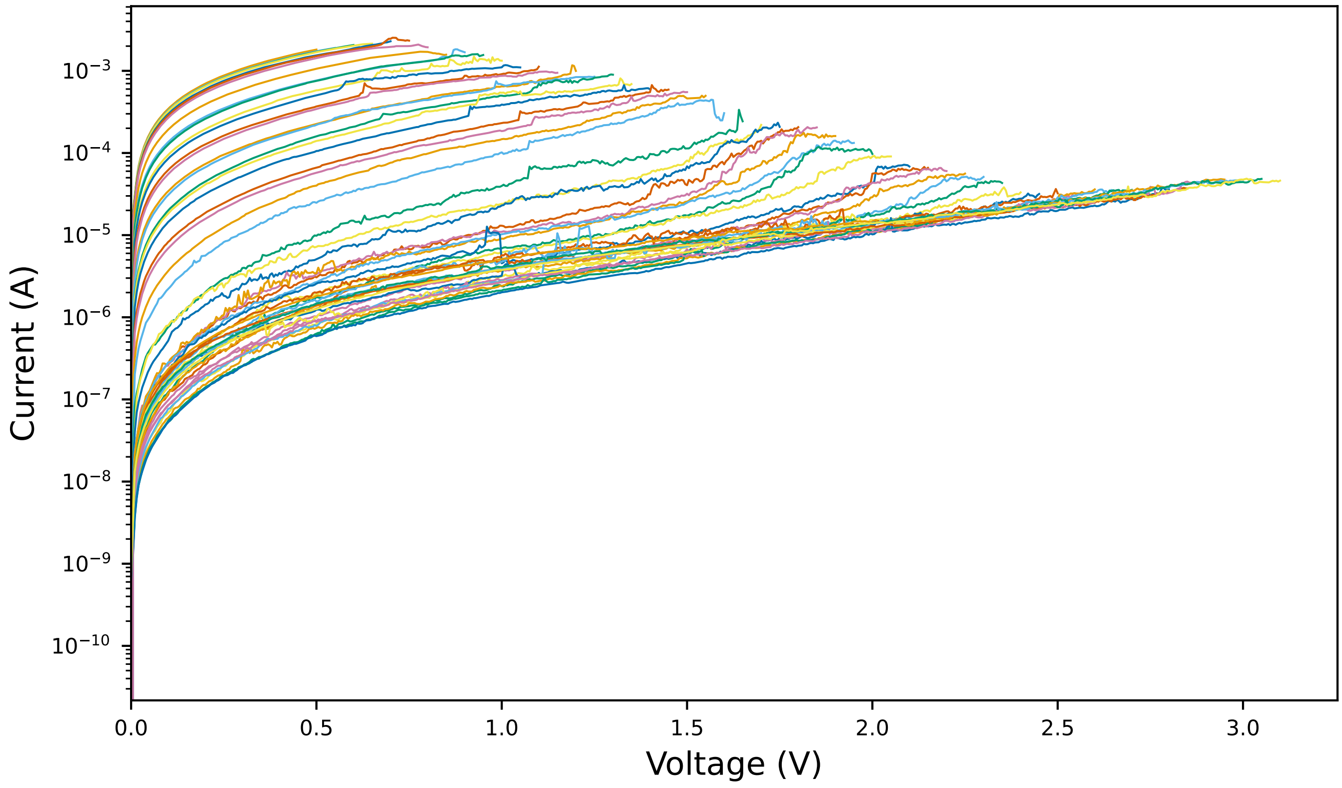

Figure S2: All I-V sweeps of the SiO_x device. Single sweeps are shown for 53 different states obtained by incrementing maximum voltage by 0.05 V. Every seventh state shares the same color.

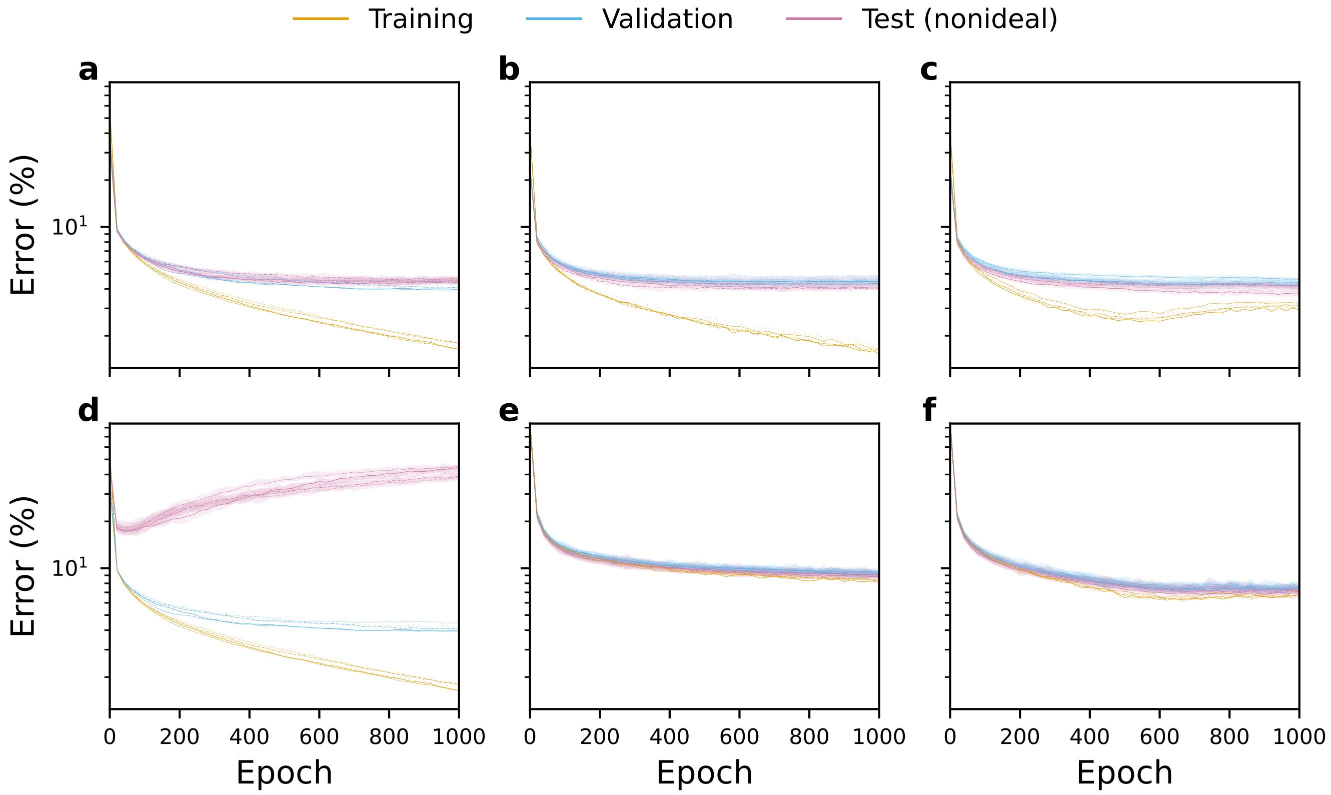

Figure S3: Training results for standard and nonideality-aware schemes when exposed to *I*-*V* nonlinearities. Panels include equivalent curves of Figure 3 for all five trained networks. **a**, **d**) Training setup 1, **b**) training setup 2, **c**) training setup 3, **e**) training setup 4, **f**) training setup 5; **a**–**c**) test setup 2, **d**–**f**) test setup 3. Curves of different networks are indicated by different line styles in each of the panels.

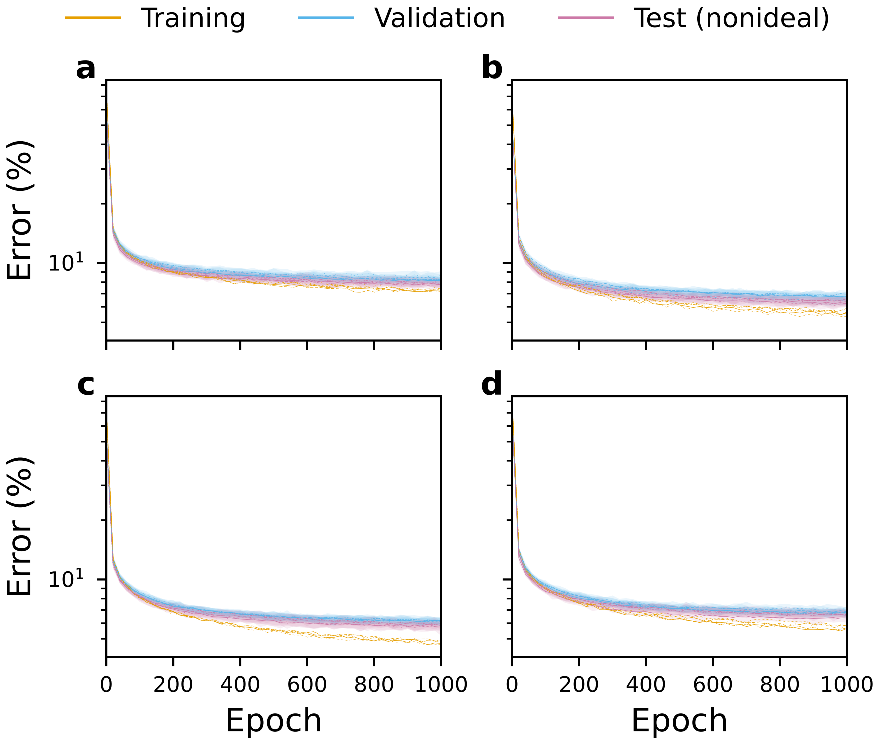

Figure S4: Training results for nonideality-aware scheme with conventional weight implementations when exposed to device-to-device variability. **a**) Training setup 6, test setup 4, **b**) training setup 7, test setup 5, **c**) training setup 10, test setup 7, **d**) training setup 11, test setup 8. Curves of different networks are indicated by different line styles in each of the panels.

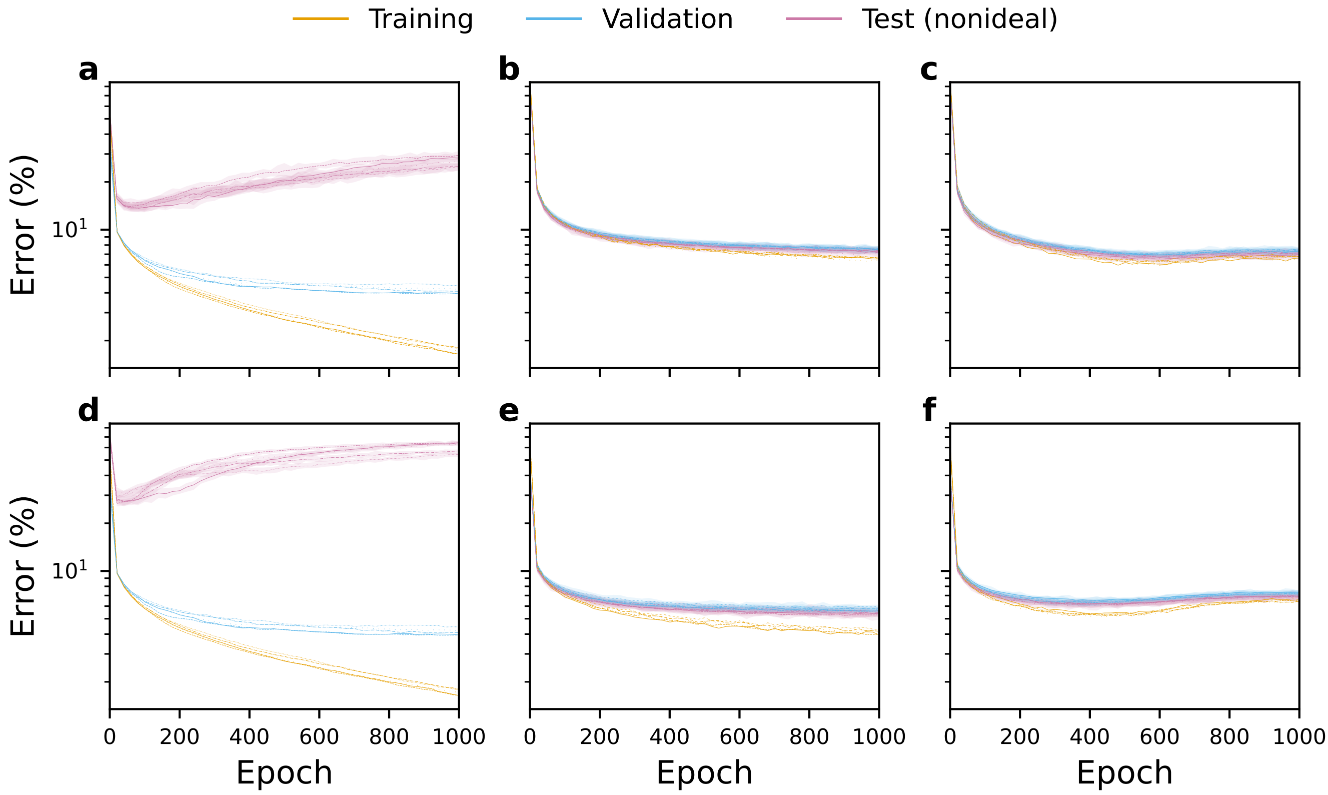

Figure S5: Training results for standard and nonideality-aware schemes when exposed to device-to-device variability. **a**, **d**) Training setup 1, **b**) training setup 8, **c**) training setup 9, **e**) training setup 12, **f**) training setup 13; **a**–**c**) test setup 6, **d**–**f**) test setup 9. Curves of different networks are indicated by different line styles in each of the panels.

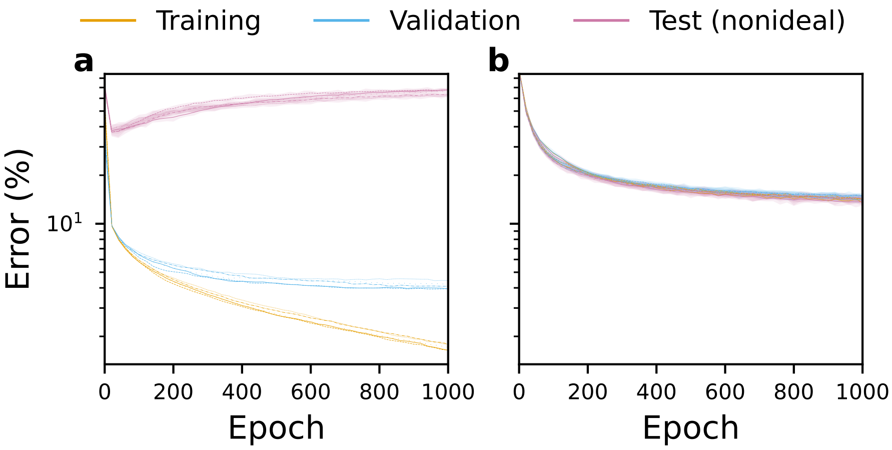

Figure S6: Training results for standard and nonideality-aware schemes when exposed to high-magnitude device-to-device variability. **a**) Training setup 1, **b**) training setup 14; test setup 10 was used in both panels. Curves of different networks are indicated by different line styles in each of the panels. Highly stochastic nonidealities like high-magnitude device-to-device variability prevent the training from overfitting to a particular set of behaviors—this is evident from how coupled training, validation and test curves are in **b**.

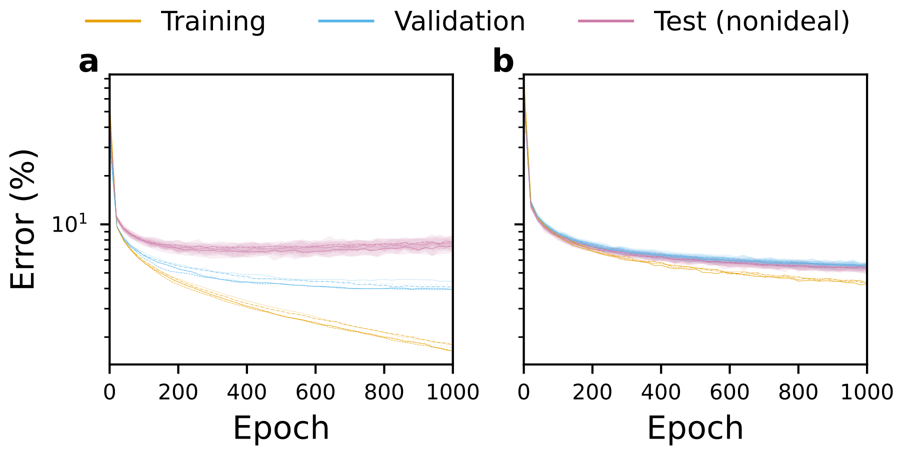

Figure S7: Training results for standard and nonideality-aware schemes when exposed to devices getting stuck at G_{off}. **a**) Training setup 1, **b**) training setup 15; test setup 11 was used in both panels. Curves of different networks are indicated by different line styles in each of the panels.

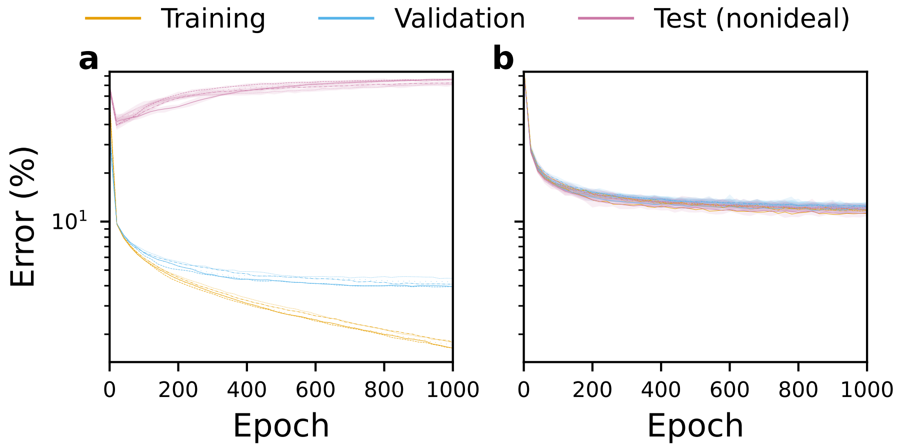

Figure S8: Training results for standard and nonideality-aware schemes when exposed to high I-V nonlinearity and devices getting stuck at G_{on}. **a**) Training setup 1, **b**) training setup 17; test setup 13 was used in both panels. Curves of different networks are indicated by different line styles in each of the panels.

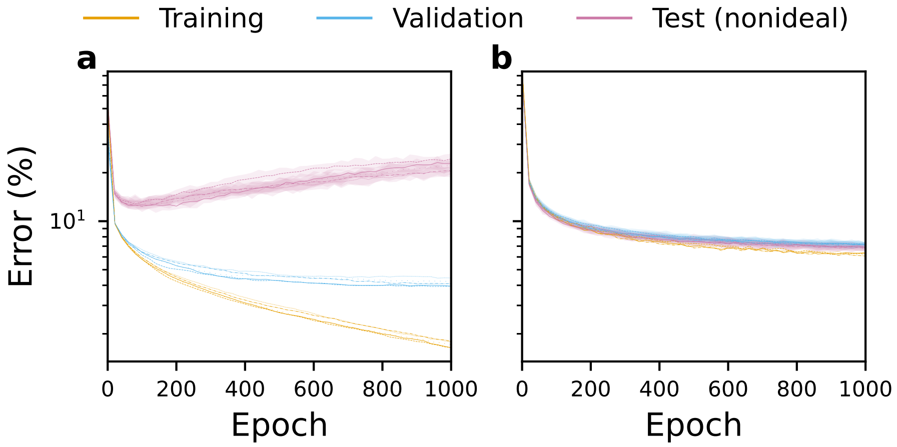

Figure S9: Training results for standard and nonideality-aware schemes when exposed to devices getting stuck. **a**) Training setup 1, **b**) training setup 16; test setup 12 was used in both panels. Curves of different networks are indicated by different line styles in each of the panels.

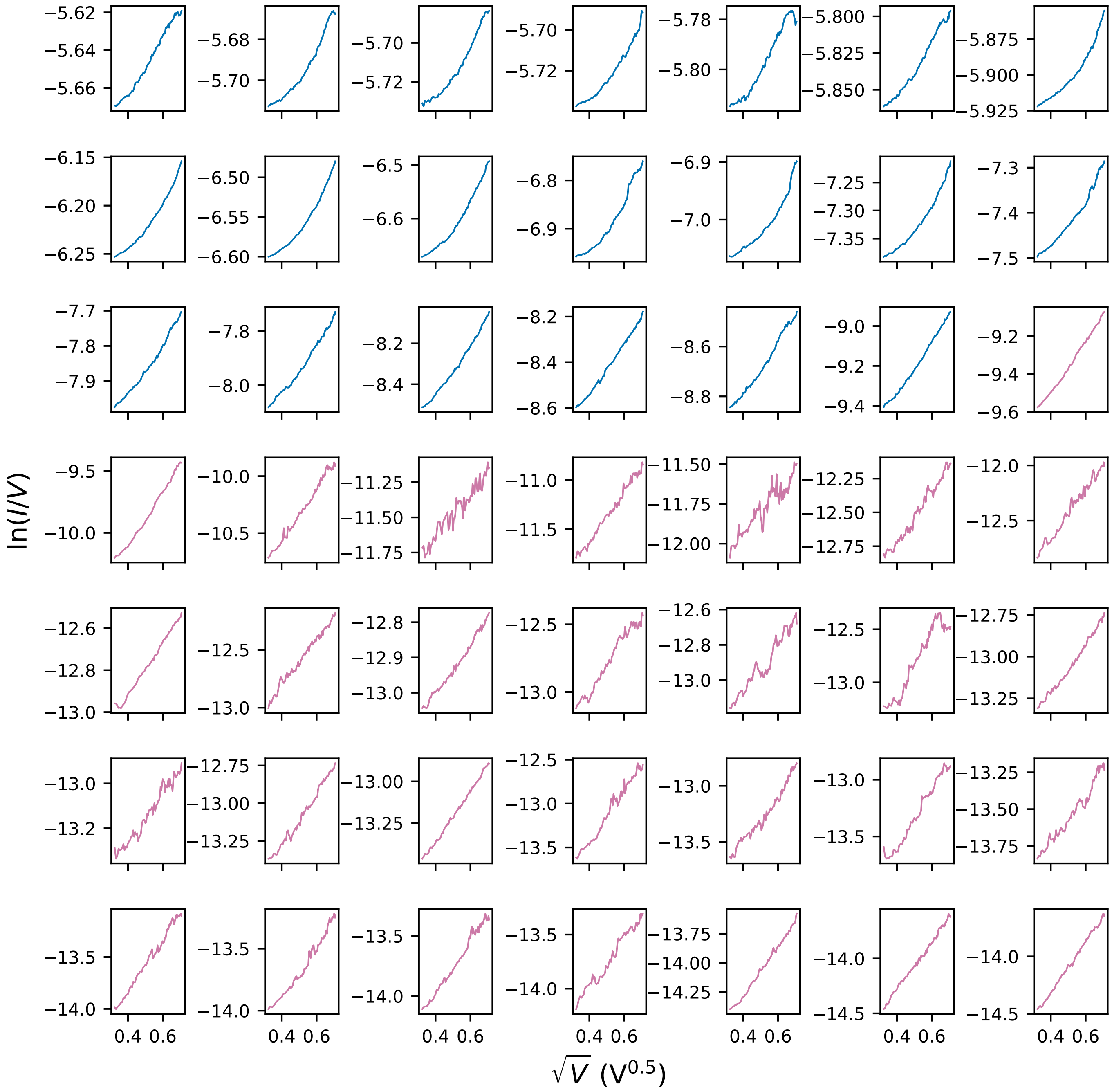

Figure S10: Poole-Frenkel plots for 49 resistance states. The plots are shown for both low-resistance (in blue) and high-resistance (in pink) states in the range from 0.1 V to 0.5 V. The inputs to logarithms are made dimensionless by using the amounts of the corresponding quantities in SI units.
